# PACE4 inhibitors and their peptidomimetic analogs block prostate cancer tumor progression through quiescence induction, increased apoptosis and impaired neovascularisation

**DOI:** 10.18632/oncotarget.2918

**Published:** 2015-02-19

**Authors:** Christine Levesque, Frédéric Couture, Anna Kwiatkowska, Roxane Desjardins, Brigitte Guérin, Witold A. Neugebauer, Robert Day

**Affiliations:** ^1^ Department of Surgery/Urology Division and Faculté de Médecine et Sciences de la Santé, Université de Sherbrooke, Sherbrooke, Québec, J1H 5N4, Canada; ^2^ Institut de Pharmacologie de Sherbrooke, Faculté de Médecine et Sciences de la Santé, Université de Sherbrooke, Sherbrooke, Québec, J1H 5N4, Canada; ^3^ Department of Nuclear Medicine and Radiobiology and Centre de Recherche du Centre Hospitalier Universitaire de Sherbrooke, Faculté de Médecine et Sciences de la Santé, Université de Sherbrooke, Sherbrooke, Québec, J1H 5N4, Canada

**Keywords:** PACE4, Multi-Leu peptide, Prostate Cancer, Proprotein convertases, Peptide inhibitors

## Abstract

Prostate cancer is the leading cancer in North American men. Current pharmacological treatments are limited to anti-androgen strategies and the development of new therapeutic approaches remains a challenge. As a fundamentally new approach, we propose the inhibition of PACE4, a member of the proprotein convertases family of enzymes, as a therapeutic target in prostate cancer. We developed an inhibitor named the Multi-Leu peptide, with potent *in vitro* anti-proliferative effects. However, the Multi-Leu peptide has not been tested under *in vivo* conditions and its potency under such conditions is most likely limited, due to the labile characteristics of peptides in general. Using a peptidomimetic approach, we modified the initial scaffold, generating the analog Ac-[DLeu]LLLRVK-Amba, which demonstrates increased inhibitory potency and stability. The systemic administration of this peptidomimetic significantly inhibits tumor progression in the LNCaP xenograft model of prostate cancer by inducing tumor cell quiescence, increased apoptosis and neovascularization impairment. Pharmacokinetic and biodistribution profiles of this inhibitor confirm adequate tumor delivery properties of the compound. We conclude that PACE4 peptidomimetic inhibitors could result in stable and potent drugs for a novel therapeutic strategy for prostate cancer.

## INTRODUCTION

The proprotein convertases (PCs) are increasingly implicated in various types of cancers, as well as cancer hallmarks. Among this family of enzymes, seven members (furin, PC2, PC1/3, PC4, PACE4, PC5/6 and PC7) process substrates at multi-basic sites, with the general consensus motif R^P4^-X^P3^-K/R^P2^-R^P1^ (X represents any amino acid) [1]. In the secretory pathway, the main function of PCs is the endoproteolytic activation of a broad spectrum of precursor proteins, including known cancer-associated proteins, such as matrix metalloproteases, adhesion molecules and growth factors and receptors [2–4]. The PCs have been extensively studied and linked to malignant phenotypes and disease progression in models of colon carcinoma [5–7], melanoma [8], cervical cancer [9], head and neck squamous carcinoma [10–12], breast [13, 14], ovarian [15, 16] and prostate carcinoma [17, 18]. Through this accumulating evidence, it is now clear that PCs are essential participants in the multi-step processes of carcinogenesis through the activation of a variety of cancer-related substrates [2–4].

What remains unclear is whether PCs are suitable therapeutic targets in cancer. Furthermore, should one or more PCs be targeted simultaneously? And are these PCs suitable targets in all types of cancers? In the case of prostate cancer, it is well understood that tumor progression is dependant on androgen signaling, and the cornerstone of pharmacological therapeutic intervention relies on androgen ablation resulting in major benefits for cancer patients. However, tumor cells eventually circumvent androgen dependency and proliferate despite castrate levels of androgens. Clearly there is a need for new therapeutic targets that could complement or follow the current anti-androgen strategies [17, 18].

Our recent work has provided significant evidence that PACE4 is a druggable target in prostate cancer and we thus devised a novel therapeutic strategy based on PACE4. Our initial observations were based on the study of tissues from radical prostatectomies, where we observed PACE4 overexpression in prostate cancer in all patients tested. In contrast, other PCs were unaffected [18]. To evaluate the importance of this observation, we turned to the study of well-established prostate cancer cell lines, namely DU145 and LNCaP cells [17, 18]. Following and initial characterization of these cell lines for their PCs expression levels, we prepared stable knockdowns for each PC expressed. Only when PACE4 was knocked down, and not the other PCs, was cell proliferation, clonogenic growth and tumor growth in xenograft mouse models decreased [17, 18].

Having provided proofs through molecular silencing studies of the involvement of PACE4 in prostate cancer progression, we then turned our attention to small-molecule pharmacological inhibitors that could be applied exogenous to these cell lines and eventually be tested *in vivo*. Unfortunately, potent and specific small molecule PC inhibitors are non-existent, with the rare exception of the recent work carried out for furin inhibitors [19, 20]. We have recently reported the development of a potent PACE4 inhibitor, named the Multi-Leu (ML)-peptide (Ac-LLLLRVKR-NH_2_) [21]. This nanomolar inhibitor is the first PACE4 inhibitor described, displaying a 20-fold selectivity for PACE4 over furin. Tested on DU145 and LNCaP cells, the ML-peptide displayed anti-proliferative inhibitory effects in cell-based assays and was shown to act through the inhibition of processing of mitogenic factors. However, the ML-peptide has not yet been evaluated under *in vivo* conditions.

While the ML-peptide shows great promise as a lead compound, it is unlikely that it would yield long lasting or potent effects *in vivo*. Indeed, peptides are rapidly metabolized or cleared, thereby significantly affecting their potential beneficial effects *in vivo*. One potential solution to this problem is to maintain the basic structure of the molecule while providing protection from proteases using peptidomimetic approaches. We therefore conducted structure-activity relationship studies to establish strategies that would improve the stability of the ML-peptide in biological systems and various substitutions in the core sequence of the ML-peptide were evaluated [22]. As with many peptides, N- and C-terminal degradation events by amino- and carboxy-peptidases are the most common and rapid to occur [22]. The priority was therefore to protect both ends of the peptide, while attempting to balance stability and pharmacodynamics. Since the C-terminal Arg of the ML-peptide inhibitor is vital to maintain inhibitor activity, we used a decarboxylated amidinobenzylamide (Amba) arginine mimetic as a replacement. The Amba substitution was previously reported at the C-terminal of furin inhibitors [19, 20] and we showed that its use in the ML-peptide led to a significant increase in stability, as well as the potency of our ML inhibitor. The N-terminal of the ML-peptide inhibitor is less problematic and its protection from aminopeptidases was accomplished by the substitution of the N-terminal leucine with a D-leucine isomer. In the present study, we combined these two modifications (D-Leu^P8^ and Amba^P1^) and evaluated the inhibitory potency of this ML-peptide analog, as an *in vivo* pharmacological inhibitor.

## RESULTS

### Peptidomimetic strategies increase the inhibitory potency and stability of the ML-peptide *in vitro*

**Figure 1:**
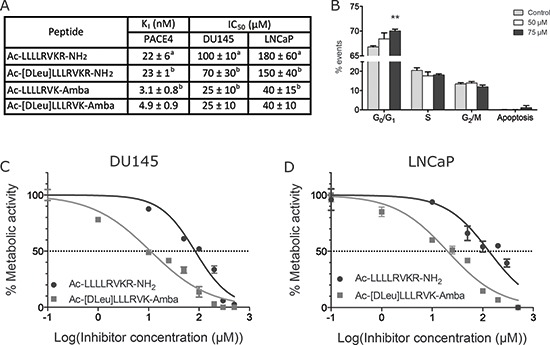
Inhibitory potency of peptidomimetic analogs **(A)** Inhibition constants (K_i_) toward PACE4 and half-inhibitory concentration (IC_50_) determined for peptidomimetic analogs *in vitro*. Footnote: ^a^ Data previously published in Levesque et al [21]. ^b^ Data previously published in Kwiatkowska et al [22]. **(B)** Cell cycle assay on LNCaP cells using the peptide Ac-[DLeu]LLLRVK-Amba. Statistical significance was established from an un-paired, two-tailed student T test. **p < 0.01. IC_50_ were calculated from MTT assays in **(C)** DU145 and **(D)** LNCaP prostate cancer cell lines. Data in the figure are mean ± SD of at least 3 independent experiments.

Peptidomimetic is an advantageous method to enhance drug-like properties of peptides inhibitors by improving their stability and biological activity. Unnatural residues were introduced in the initial scaffold Ac-LLLLRVKR-NH_2_ and the resulting peptidomimetic analogs are presented in Figure 1A. The introduction of the stereoisomer D-Leu in the position P8 did not affect the affinity of ML-peptide for PACE4 *in vitro*. On the other hand, both peptides Ac-LLLLRVK-Amba and Ac-[DLeu]LLLRVK-Amba displayed significant improvements in inhibitory potency, demonstrating that the introduction of the conformationally-restricted moiety Amba within the PCs recognition motif increased its affinity for PACE4. The peptide resulting from combination of both modifications Ac-[DLeu]LLLRVK-Amba is a low nanomolar PACE4 inhibitor *in vitro* (K_i_ 4.9 ± 0.9 nM) with a 4-fold increase in potency when compared to a control ML inhibitor (K_i_ 22 ± 6 nM). When tested in cell based assays, the peptide Ac-[DLeu]LLLRVK-Amba exhibited strong antiproliferative properties on both DU145 and LNCaP prostate cancer cell lines, with IC_50_s of 25 ± 10 μM and 40 ± 10 μM respectively (Figure 1A,1C–1D). A cell-cycle analysis performed on LNCaP cells treated with 50 and 75 μM of Ac-[Dleu]LLLRVK-Amba peptide reveals a dose-response G_0_/G_1_ cell cycle arrest along with increased apoptotic events (Figure 1B). Interestingly, blockade of the cell cycle from G_0_/G_1_ through S phase and induction of apoptosis is a phenotype that can be associated with growth factor withdrawal in cell culture assay, suggesting that PACE4 substrates in LNCaP cells enhance proliferation and survival capabilities. Furthermore, a similar result was previously obtained with the ML-peptide treated LNCaP cells [21]. However, doses of up to 200 μM were required for this unmodified peptide [21]. This demonstrates that the Ac-[Dleu]LLLRVK-Amba analog is more potent and/or more stable in this cell assay. Since the same cell cycle parameter changes were observed with the Ac-[Dleu]LLLRVK-Amba and the ML-peptide, it is likely that the observed anti-proliferative effects occur through the same mechanisms of action.

**Figure 2:**
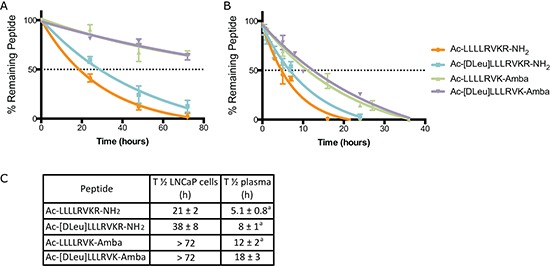
Stability of peptidomimetic inhibitors **(A)** LNCaP cells *in vitro* assay and in **(B)** plasmatic *ex vivo* stability assay demonstrate increased stability of peptidomimetic analogs. **(C)** Half-life (T_½_) was calculated from stability assays and compared to the unmodified ML-peptide. Data in the figure are mean ± SD of 2 independent assays. Footnote: ^a^ Data previously published in Kwiatkowska et al [22].

Beside a gain in inhibitory potency (i.e., improved K_i_ values), peptidomimetic strategies also aim at improving peptide stability. In cell-based assays using LNCaP cells, the stability of the ML-peptide and its peptidomimetic analogs were compared (Figure 2A). Half-life (T_½_) of 21 ± 2 h for ML-peptide, 38 ± 8 h for Ac-[DLeu]LLLRVKR-NH_2_ peptide, and > 72 h for both Ac-LLLLRVK-Amba and Ac-[DLeu]LLLRVK-Amba peptides were observed. Interestingly, more that 90% of each analog was intact when incubated with complete media only (data not shown), indicating that degradation occurs mostly from cell-derived proteases rather than serum constituents in this assay. These improvements in stability, along with the increased affinity for PACE4 are both important factors that explain the greatly increased anti-proliferative potency observed with the peptidomimetic analogs as compared to the ML-peptide in a 72 h cell proliferation MTT assay (Figure 1C–1D). The compound stability was then assayed *ex vivo* in mouse plasma, which is closer to representative *in vivo* conditions (Figure 2B–2C). For each analogs, the *ex vivo* stability half-life was shorter than 24 h, demonstrating that degradation occurs at an increased rate in plasma as compared with LNCaP cell line. Nonetheless, the introduction of peptidomimetic modifications results in significantly increased peptide stability with T_½_ up to 18 ± 3 h for the Ac-[DLeu]LLLRVK-Amba peptide, a 3.2-fold improvement when compared to the ML-peptide (T_½_ 5.1 ± 0.8 h).

### Peptidomimetic strategies increase the inhibitory potency in LNCaP xenograft assays

**Figure 3:**
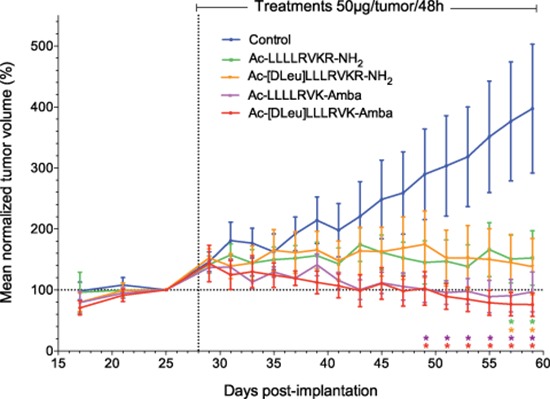
*In vivo* inhibitory potency of peptidomimetic inhibitors Inhibitory peptides display significant antiproliferative effects on LNCaP xenografts *in vivo* when administered directly into the tumors. Statistical significance was established from an unpaired two-tailed student T test. *p < 0.05; *n* = 9–10 tumors per group. Data in the graph are mean ± SEM of normalized tumor volume at day 25.

In order to assess the *in vivo* potency of the ML-peptide and its analogs, LNCaP xenograft experiments were performed with each peptide (Figure 3). First, intra-tumoral administration of compounds was performed to ensure that effects on tumor growth are observable *in vivo*, by limiting biodistribution conditions. Following administration of inhibitors at a dose of 50 μg/tumor per 48 h period, all peptides displayed significant inhibitory properties on LNCaP xenograft proliferation. Vehicle-treated tumors had a steady growth rate, with mean tumoral volumes of 400 ± 100% at day 59 compared to day 25, while the ML-peptide (Ac-LLLLRVKR-NH_2_) and its analog Ac-[Dleu]LLLRVKR-NH_2_ displayed inhibitory effects with reduced tumor volumes of 150 ± 40% and 140 ± 50% respectively (Figure 3). The ML-peptide analogs, Ac-LLLLRVK-Amba and Ac-[Dleu]LLLRVK-Amba, both containing the Amba arginine mimetic modification displayed enhanced inhibitory potencies, with tumor volumes of similar size at the end of the experiment when compared to the initial volumes at the time of first treatment at day 25 (Ac-LLLLRVK-Amba: 100 ± 30%; Ac-[Dleu]LLLRVK-Amba: 80 ± 20%). Interestingly, the peptide Ac-[Dleu]LLLRVK-Amba is the only inhibitor for which a decrease tumor volume at completion of the experiment was observed. This result demonstrates the effectiveness of the ML-peptide on LNCaP tumor growth but also the relevance of peptidomimetic modifications in enhancing the inhibitory potency *in vivo.*

### Pharmacokinetic (PK) profile of the Ac-[DLeu]LLLRVK-Amba peptide

**Figure 4:**
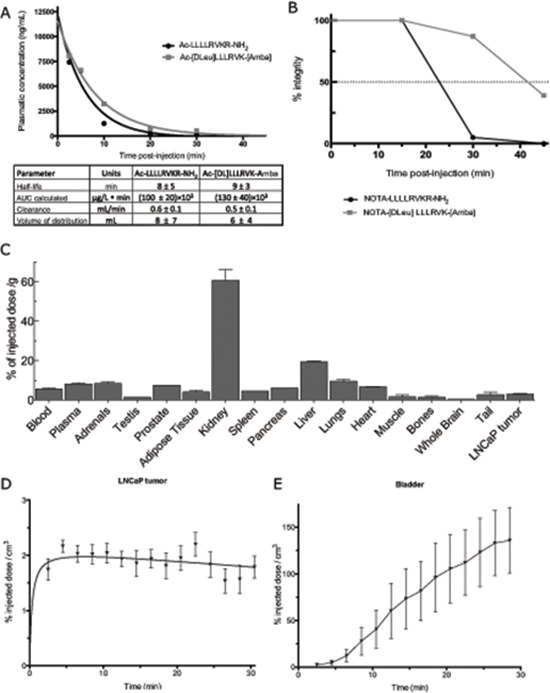
Pharmacokinetic profile of the peptide Ac-[DLeu]LLLRVK-Amba **(A)** Pharmacokinetic profile of the ML-peptide and Ac-[DLeu]LLLRVK-Amba peptide determined by mass spectrometry analyses from intravenous 2 mg/kg dose. **(B)** *In vivo* stability assay using ^64^Cu-radiolabeled NOTA peptides carried out at 0, 15, 30 and 45 minutes post-injection. **(C)** Biodistribution profile of ^64^Cu-conjugated peptide expressed in percentage of total dose per gram of tissue. Tissue-specific kinetic of distribution for **(D)** LNCaP tumor and **(E)** bladder determined from image-reconstruction of 30 minutes scans.

As our intention is to test these compounds through an intravenous route of administration, the next step was to examine the pharmacokinetic profile of the ML-peptide and its stable peptidomimetic analog Ac-[DLeu]LLLRVK-Amba. Plasmatic concentration of peptide following a systemic 2mg/kg dose were quantified by mass spectrometry (Figure 4A). No significant difference was established in PK parameters for both the ML and the Ac-[DLeu]LLLRVK-Amba peptides. The *in vivo* plasma half-lives of both compounds were short (ML: 8 ± 5min; Ac-[DLeu]LLLRVK-Amba: 9 ± 3min) with a total clearance rate of 0.6 ± 0.1 mL/min and 0.5 ± 0.1 mL/min for the ML and the Ac-[DLeu]LLLRVK-Amba, respectively. However, the high volume of distribution (ML: 8 ± 7 mL; Ac-[DLeu]LLLRVK-Amba: 6 ± 4 mL) suggests high tissue penetration properties for both peptides.

*In vivo* stability and biodistribution were then evaluated using a ^64^Cu/NOTA (1,4,7-triazacyclononane-triacetic acid) bifunctionnal chelating group conjugated analog, following an approach that we have previously described [23]. The NOTA moiety displays a high affinity for metal ions thus allowing the labeling of a peptide with ^64^Cu, a low energy positron-emitter (E_β_ 656 kev). The NOTA bifunctionnal chelating group was introduced in the N-terminal of the peptide in order to avoid interference with the C-terminal basic residues that are key to PC recognition. Comparison of the K_i_ of the NOTA derived peptides with those of the original peptides revealed no difference in PACE4 affinity between peptides Ac-[DLeu]LLLRVK-Amba (K_i_: 4.9 ± 0.9 nM, Figure 1) and NOTA-[DLeu]LLLRVK-Amba (K_i_: 6 ± 2 nM) and between the ML-peptide and its NOTA-labeled counterpart [23]. The *in vivo* stability of ^64^Cu/NOTA-[DLeu]LLLRVK-Amba was then assayed and an intact peptide was quantified at 0, 15, 30 and 45 minutes post-intravenous administration (Figure 4B). When compared to the ^64^Cu/NOTA-LLLLRVKR-NH_2_ peptide, ^64^Cu/NOTA-[DLeu]LLLRVK-Amba demonstrated increased stability properties, being 87% intact compared to 5% for control peptide, 30 minutes post-injection. No free ^64^Cu was detected in serum (data not shown) consistent with known strong interactions between ^64^Cu and the NOTA moiety [24]. Using ^64^Cu/NOTA-[DLeu]LLLRVK-Amba peptide, the biodistribution profile was determined 20 minutes after intravenous administration from radioactivity counts in dissected organs and reported as percentage of administered dose per weight of tissue (%/g) (Figure 4C). The radiopeptide was predominantly detected the kidney (60 ± 8%/g), suggesting important renal elimination. This data provides evidence that the short half-life observed in Figure 4A is likely to be due to a rapid clearance of the peptide *in vivo*, rather than its metabolic processing when injected intravenously. Indeed the plasma half-life *ex vivo* was shown to be in hours (Figure 2C) and peptide was shown to be stable up to 30 minutes post-injection (Figure 4B). Aside from the kidney, the ^64^Cu/NOTA-[DLeu]LLLRVK-Amba peptide was also present in other organs, as well as in LNCaP tumors (2.9 ± 0.5%/g) (Figure 4C). This biodistribution profile confirms the organ-penetration properties of the peptide, as initially suggested by high volume of distribution measured (Figure 4A). This data is consistent with our previous observations [21, 22] and could be due to the lipophilic nature and/or the cell-penetration properties of the ML-peptide and analogs. Radiolabeling of peptides with ^64^Cu, a low energy β-emitter allows for high resolution PET (Positron emission tomography) imaging. Thus, to understand the kinetic of distribution of the Ac-[DLeu]LLLRVK-Amba peptide, PET imaging was performed using its radiolabeled counterpart and quantified in LNCaP tumor (Figure 4D) and bladder (Figure 4E) over time. The peptide rapidly penetrated into the tumor after intravenous administration and the signal remained stable in this tissue over a 30 minutes period, again suggesting excellent tissue penetration properties. In contrast, the observation of radioactive signal into the bladder revealed a steady renal elimination rate of the peptide post administration, which is a common mechanism of elimination for small peptides [25]. Overall, the pharmacokinetic and biodistribution profile of the Ac-[DLeu]LLLRVK-Amba peptide and its radiolabeled counterpart demonstrates that although this inhibitor is rapidly cleared from plasma by renal excretion, it is distributed and retained in the tumor, providing solid evidence that the stable peptidomimetic analog reaches its pharmacological target.

### The Ac-[DLeu]LLLRVK-Amba analog inhibits tumor progression *in vivo*

**Figure 5:**
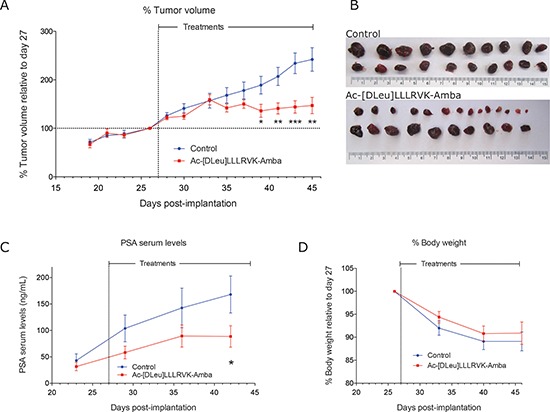
Systemic administration of the peptide Ac-[DLeu]LLLRVK-Amba *in vivo* Tumor progression following 2 mg/kg IV treatments was established from **(A)** tumor volume measurements and **(B)** LNCaP treated tumor harvested at the completion of experiment compared to control vehicle-treated tumors. **(C)** PSA serums levels quantification. **(D)** Body weight of mice during the experiment decreased in both group as tumor were progressing. Statistical significance was established from unpaired two-tailed student *T* tests. *p < 0.05; **p < 0.01; ***p < 0.001; *n* = 11 mice per group. Data in the graph are mean ± SEM of normalized tumor volume at day 27.

The pharmacologic effects of the potent and stable Ac-[DLeu]LLLRVK-Amba peptide analog were then studied *in vivo* following daily 2 mg/kg *iv* dose in the LNCaP xenograft mouse model. Tumor progression was monitored by tumor volume measurements and PSA (prostate-specific antigen) serum levels (Figure 5A–5C). The systemic administration of the Ac-[DLeu]LLLRVK-Amba significantly inhibited tumor progression, with tumor volumes reduced by 60% over 18 days of treatments when compared to vehicle-treated animals. PSA levels followed the same pattern with serum levels averaging 170 ± 40 ng/mL for control and 90 ± 20 ng/mL for treated animals at the completion of the experiment, which represent a reduction of 47%. These data demonstrate that although only a fraction of initial dose reaches the LNCaP tumor as determined by our PK studies, this proportion is sufficient to trigger a pharmacologic effect. To monitor the toxic effects of peptide administration, body weight was measured weekly (Figure 5D). Both treatment and control groups display similar weight loss of 10%, which suggests that tumor growth was mostly responsible for this decrease in body weight rather than the treatment itself.

### The Ac-[DLeu]LLLRVK-Amba peptide induces cell quiescence and apoptosis and hinders tumor vascularisation *in vivo*

**Figure 6:**
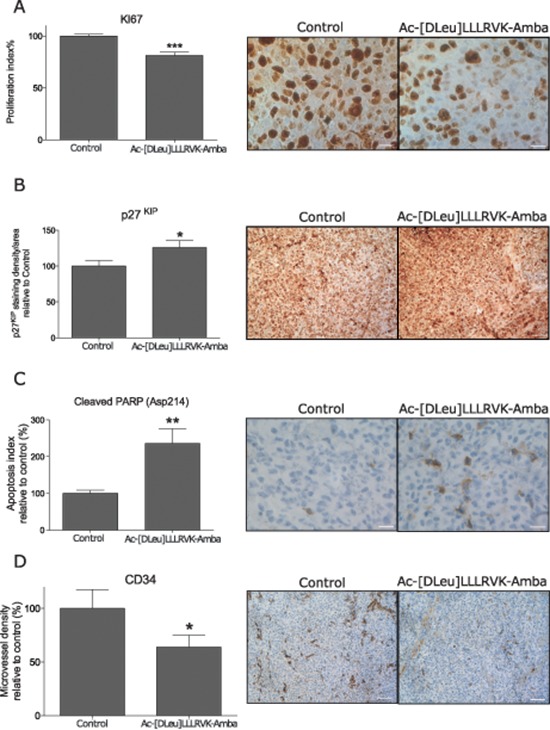
Immunohistochemistry analyses on harvested tumors Immunohistochemistry analyses for **(A)** proliferation marker Ki-67 and **(B)** quiescence marker p27^KIP^ reveals a cell cycle arrest in the Ac-[DLeu]LLLRVK-Amba treated tumors. **(C)** Apoptosis index was evaluated from cleaved PARP at Asp214 marker and **(D)** micro vascularization was assessed from CD34 immunostaining. Statistical significance was established from unpaired two-tailed student *T* tests. *p < 0.05; **p < 0.01; ***p < 0.001; *n* = 12–15 tumor per group, and 3 to 5 area were evaluated for each tumor. Data in histograms are mean ± SEM and pictures are representative area. Scale bar represent 25 μm (Ki-67; PARP Asp214) and 100 μm (p27^KIP^; CD34).

At the completion of the experiment, tumors were harvested and histological analyses were performed using well-established biomarkers to determine the effects of the peptide on cell proliferation and progression through cell cycle within tumors. Immunostaining for Ki-67, a proliferation marker, revealed a significant 20% reduction of cells progressing through cell cycle, with a relative count of 81 ± 3% of Ki-67^+^ cells from treated animals when compared to control tumors (100 ± 2%) (Figure 6A). The p27^KIP^ immunostaining in tumors, which is indicative of quiescent cells, demonstrated a significant increase among treated tumors with a relative staining of 130 ± 10% versus 100 ± 8% for control tumors (Figure 6B). From the cleaved PARP (Asp214) apoptosis marker immunostaining, a significant increase in the rate of cells under apoptosis was observed (Figure 6C). From the baseline 2.4 ± 0.2% of apoptotic cells within control tumor, this rate increased to 6 ± 1% in treated tumors, which represent a 250% increase. The decreased cell proliferation rate and increased in cell quiescence along with the induction of apoptosis in tumors of treated animals is consistent with data obtained from *in vitro* cell cycle assay of LNCaP cells treated with peptide Ac-[DLeu]LLLRVK-Amba (Figure 1B), indicating that the molecular effects of this peptide on LNCaP cells *in vitro* are preserved *in vivo.* The far-reaching effects that result from PACE4 inhibition are consistent with the role of PACE4 as a hub protein for the activation of cancer promoting factors in prostate cancer. Since LNCaP xenografts are well known for their high rate of angiogenesis, histological analysis was performed with the endothelial cell marker CD34 (Figure 6D). Tumor micro-vascularization was significantly decreased in Ac-[DLeu]LLLRVK-Amba treated animals when compared to controls with a relative microvessel count of 60 ± 10% compared to 100 ± 20% in control animals. Various pro-angiogenesis factors require processing by the PCs (e.g.: vascular endothelial growth factor, basic fibroblast growth factor, transforming growth factor-β, platelet-derived endothelial growth factor).

## DISCUSSION

There is increasing evidence for the involvement of PCs in carcinogenesis and disease progression [2, 3, 26–29] and there is now little doubt for their importance in cancer. However, an understanding of the specific steps where PCs intervene and their temporal relationships are yet to be fully deciphered. What is known so far, is that PCs act by cleaving cancer-related substrates, such as growth factors, receptors, proteases and adhesion molecules, mostly in activating functions. Although not necessarily expected, aberrant PCs expression patterns have also been observed in various cancers [2, 3, 26–29], suggesting a fine-tuned coordinated regulation of PCs with these cancer-associated substrates in order to sustain their processing. This appears to be the case for PACE4, which was observed to be overexpressed in prostate cancer tissues [18]. PACE4 up-regulated expression has been also been observed in breast [13] and ovarian [16] cancers. While the molecular mechanisms involved are certainly worthy of deeper investigations, the question remains as to whether PACE4 can be considered as a therapeutic target or even a druggable target. In itself, up-regulation of PC gene expression does not provide evidence for or against suitability as a drug target. To test that hypothesis, we have previously used prostate cancer cells lines to demonstrate that blocking PACE4 expression levels results in significant anti-proliferative effects and reduced tumor size in xenograft animal models [17]. This effect was specific to PACE4 and not observed when blocking other PCs expressed in these cell lines, including furin and PC7 [17]. Our data provide strong evidences that PACE4 functions are intimately related to prostate cancer progression and further suggest PACE4 as a druggable target. This observation has motivated further development of pharmacological inhibitors of PACE4 for *in vivo* use.

We first described a novel high affinity and selective PACE4 inhibitor, namely the ML-peptide, (Ac-LLLLRVKR-NH_2_) [21], which is 20 fold more potent for PACE4 than furin and displays anti-proliferative effects in the prostate cancer cell lines DU145 and LNCaP [21]. We believe this peptide can be used as a lead for the design of a more stable compound for *in vivo* use. From a clinical point of view, peptides offer numerous advantages, such as their high affinity and specificity for their biological target, while generally being less toxic, thus making them excellent drug candidates [30, 31]. Recent advancements in medicinal chemistry have overcome the traditional limitations of therapeutic peptides such as poor pharmacokinetic properties, limited membrane penetration and metabolic stability [32, 33]. Although peptides were traditionally not considered as potential drugs, it is now estimated that the market size of peptide therapeutics is growing twice as fast as any other pharmaceutical [32].

In order to test the effects of the ML-peptide *in vivo*, we first decided to maximize the chances of success by using peptidomimetic strategies to increase its stability in biological systems. Indeed, we previously observed that the ML-peptide is prone to rapid degradation from plasma-derived exopeptidases as typically observed for naturally-occurring amino acids based peptides [22]. Substitution with unnatural amino acids as a strategy allowed for important gains in both inhibitory potency and peptide metabolic stability [22]. The sequential addition of the Amba^P1^ and the DLeu^P8^ residues in the ML-peptide enhanced peptide stability (Figure 2). The Amba^P1^ improved inhibitory potency and metabolic stability, while the DLeu^P8^ only contributed to peptide overall stability. Not only does it prevent attack from aminopeptidases, but most likely protects from ubiquitous leucyl aminopeptidases [34] These data are consistent with the previous observations that degradation of the ML-peptide primarily occurs from both termini ends of the peptide [22].

Our data confirms that the peptidomimetic analog Ac-[DLeu]LLLRVK-Amba resulting from the Amba^P1^ and the DLeu^P8^ substitutions displayed increased inhibitory potency both *in vitro* and *in vivo* (Figures 1–3) consistent with the increase in its stability profile and the addition of Amba moiety which promotes tight binding properties of the inhibitor [35]. When administered directly into the tumors of LNCaP xenografted mice, all analogs displayed significant inhibitory effects *in vivo*, but the Ac-[DLeu]LLLRVK-Amba peptide resulted in the most important inhibitory response. This was the first data to demonstrate that if these peptides can reach their tumor target, they should have effects on tumor progression.

In order to evaluate whether the systemic administration of PACE4 inhibitors would provide sufficient effector molecules to the target, the pharmacokinetic profiles of the highly potent and stable analog Ac-[DLeu]LLLRVK-Amba was established and compared to its unmodified counterpart. Although the peptide Ac-[DLeu]LLLRVK-Amba has an increased stability profile compared to the ML-peptide, both molecules displayed similar pharmacokinetic parameters, with half-lives in the minutes range and rapid clearance kinetics (Figure 4). Further characterization of the pharmacokinetic and distribution demonstrated important kidney clearance for the peptidomimetic analog. Indeed, although this analog is intact 30 minutes following administration, kidney clearance accounts as the most important elimination mechanism, rather than metabolic degradation. Further optimization of the ML-peptide and analogs pharmacokinetics to circumvent rapid kidney clearance would increase plasmatic half live and should increase the pharmacologic response to PACE4 inhibitors. Common strategies to inhibit glomerular filtration of peptide compound includes conjugation with large polymer, such as polar polyethylene glycol [36]. However, such modifications strongly affect cell-penetration properties of the ML-peptide [21] and therefore would influence the *in vivo* inhibitory potency of the compound. Alternative strategies such as binding to albumin should be explored [36].

In spite of the rapid clearance rates, it was also observed that the PACE4 peptide inhibitors reach the tumor rapidly and that the local concentration remains stable even after a 30 minutes post-injection period (Figure 4). This is consistent with our recent data using PET-imaging techniques which clearly shows that ML PACE4 peptide inhibitors reach the tumor target and accumulate in these cells through a PACE4 dependent mechanism [23]. While only a fraction of the ML-peptide reaches the tumor in the LNCaP xenograft model, this local concentration is sufficient to induce a pharmacological response. Indeed, the systemic administration of the peptide Ac-[DLeu]LLLRVK-Amba significantly inhibited tumor progression (Figures 5–6). Interestingly, the effects of the systemically administered PACE4 inhibitory peptides on tumor progression closely replicate the phenotype observed with the PACE4 knockdown in LNCaP and DU145 cells previously described [17]. In this model, tumor growth rates observed in a xenograft assay were significantly lower when compared to the control. At the end of the experiments, tissue characterizations were performed. Immunohistochemistry of PACE4 knockdown tumors revealed reduced Ki-67 (proliferation marker), higher p27^KIP^ (quiescence marker) and cleaved PARP (Apoptosis marker) levels along with a decrease in tumor microvascularization, as seen from CD34 immunostaining. The similarity between previously obtained PACE4 molecular inhibition, (i.e., PACE4 shRNA-silenced LNCaP cells) and the pharmacologic inhibition obtained from the systemic administration of the ML-peptide analog, provides evidence for the use of PACE4 peptidomimetic analogs as a potential drug for a novel therapeutic approach for prostate cancer. We also conclude that whether PACE4 is silenced through molecular interference or inhibited through exogenous pharmacological inhibitors, the tumoral phenotype demonstrates that PACE4 has a key role in regards to cell proliferation, survival and tumor progression in prostate cancer, most likely through the sustained processing of key mitogenic and pro-survival substrates.

Sustained activation of growth signaling pathways represent an important feature of disease progression for prostate cancer [37]. In advanced clinical stages of prostate cancer, growth factors promote cell proliferation and survival independently of androgen stimulation [37]. Furthermore, disease progression is closely related to angiogenesis, mediated primarily by the vascular endothelial growth factor family, which promotes routes for cell migration and ensures neovascularization of distant sites [38]. Previous attempts have been made to individually target growth factors networks and some emerging therapeutics currently under clinical trials target the vascular endothelial growth factor network, the platelet-derived growth factor receptor or the insulin-like growth factor axis [39, 40]. However, targeting individual pathways have shown some drawbacks due to the facts that growth signaling usually acts in a complex synergistic network [37, 39, 41, 42] with many redundancies. The proteolytic processing by PCs represents an upstream feature for proliferation-associated proteins that use or are part of these signaling pathways. Therefore, targeting PACE4 in prostate cancers could provide a more effective measure to counter the combined effects of multiple proliferation factors. Furthermore, up-regulation of PACE4 has been reported for every stages of the disease, thus demonstrating its involvement throughout the tumor progression timeline [18] and further suggests that early intervention to inhibit PACE4 may also be desirable. Although PACE4 is a key protein in prostate cancer and can also be considered as a hub protein in cancer cells [2, 18], its function in normal tissue homeostasis is of some concern. Indeed, targeting PACE4 could also have deleterious effects when inhibited in normal cells. However, the evidence available suggests that redundancies, by other PC family members in normal tissue, would compensate for any vital functions. For example, the full PACE4 knockout mouse phenotype is viable with no apparent defects [43]. It is therefore expected that PACE4 inhibition through systemically administered inhibitors would result upon limited toxicity.

In conclusion, the present study demonstrates that PACE4 peptidomimetic inhibitors result in stable and potent compounds whose systemic administration have an impact on prostate cancer cells. We can now envisage further studies to develop a PACE4 based therapeutic strategy for prostate cancer.

## MATERIALS AND METHODS

### Peptide synthesis

Inhibitors containing the Arg^P1^ residue were obtained manually by solid-phase peptide synthesis on a polystyrene resin as previously described [22]. Inhibitors modified with the Amba residue were obtained manually by a combination of solid-phase peptide synthesis and solution synthesis, as detailed [22]. The 1,4,7-triazacyclononane-triacetic acid (NOTA) peptide conjugate was obtained on the basis of the strategy developed by Guérin *et al* [44] using a combination of solid phase approach and solution synthesis, as described in Supplementary S1 information. All peptides were purified by reverse phase HPLC and compound identification and purity was assessed by analytical HPLC. High-resolution mass spectrometry was used to confirm the identity of the pure products. Physicochemical properties of peptides along with additional information on peptides synthesis are presented in supplementary material.

### Enzymatic assays

Inhibition constants (K_i_) were determined using human recombinant and soluble PACE4 and furin produced in S2 insect cells and purified as previously described [45]. The enzymatic assays were performed as described [21].

### Cell culture and proliferation assays

Cell lines were obtained from ATCC and maintained in RPMI-1640 5% FBS (DU145) and 10% FBS (LNCaP). For MTT proliferation assays, peptides were added 24 hours after seeding in 96-well plates as previously described and incubated 72 hours prior to addition of MTT reagent (Sigma-Aldrich, Canada) at final concentration of 1 mg/mL [21, 22]. Formazan salt was solubilized with 100 μL Isopropanol: HCl (24:1N) and metabolic activity was normalized relatively to vehicle-treated cells (Sterile bidistilled water). IC_50_ were determined using Prism 5.0 (GraphPad Software, USA).

### Cell cycle analyses

8 × 10^5^ cells LNCaP cells were seeded in 10 cm dish and peptides were added 24 hours after along with fresh media. To offset peptide degradation, media and peptide were changed after a period of 48 hours. Cell were harvested with trypsin after 72 hours and fixed with ethanol prior to DNA staining with propidium iodine as previously described [18, 21]. Acquisitions were carried using a FACScan cytometer (Becton Dickinson, USA) equipped with a 15 mW argon ion laser tuned at 488 nm. A minimum of 10,000 gated events per sample were acquired. Forward and side scatter signals were used to establish the live gate to exclude debris and cell clumps and a second live gate was set using the FL3-A and FL3-W parameters of the doublet discrimination module, allowing single cell measurements. The percentages of cells in different phases of cell cycle were calculated by ModFit software (Verity Software House, USA).

### Stability assays

LNCaP cell-based stability assays were performed in Poly-(L)lysine coated 96-wells plates seeded at a density of 1,500 cells/wells. 100 μg of peptides were added 24 hours after plating without media change. Media was collected and mixed with 5 μL of trifluororacetic acid 5% to stop peptide degradation. *Ex vivo* plasmatic stability assays were performed as previously described [22]. Briefly, compounds (0.5 μg/μL) were incubated with CD1 mouse plasma from mixed-sex animals collected with heparin sodium (Novi, USA). Reaction was stop by addition of 150 μL guanidine HCl and 300 μL of acetonitrile and peptides were extracted by centrifugation. For both *in vitro* and *ex vivo* stability assays, peptide stability was assessed from reverse phase HPLC and percentage of remaining peptide was calculated from area under curve and normalized to time 0.

### Pharmacokinetic assays

To determine pharmacokinetic profiles of peptides, a single dose of 2 mg/kg was administered into systemic circulation from tail vein of CD1 mice (*n* = 4 animal/peptide). Blood was micro sampled on individual mouse at time 2.5, 5, 10, 20, 30 and 60 minutes. Plasma was collected by centrifugation and proteins were cleared from plasma using acetonitrile precipitation and supernatant was filtered using 0.2 μm PVDF centrifugal device (Canadian Life Science, Canada). Sample were analysed at PhenoSwitch Bioscience (Sherbrooke, Canada) by LC-MS/MS on a TripleTOF 5600 mass spectrometer (ABSciex, USA) equipped with DuoSpray source. Further information on mass spectrometry analyses and parameters used are presented in supplementary material.

### Radiolabeled compound biodistribution and TEP-scan derived time-activity curve

Peptide was labeled with ^64^Cu as previously described [23]. For stability studies, the ^64^Cu-radiolabeled-peptide reconstituted in PBS (20–30 MBq; 500–800 μCi; 100 μL) was injected to isoflurane-anaesthetized CD1 mice through the tail vein. Blood was collected and analysed with quantitative TLC procedures 15, 30 and 45 minutes post-injection. To perform biodistribution studies, radiolabeled peptide (400–900 kBq; 10–25 μCi; 100 μL) was administered to isoflurane-anaesthetized CD1 mice through caudal vein injection. Organ were collected 15 minutes post-injection from CO_2_ inhalation euthanized animals and were washed, weighted and radioactivity was measured in a gamma counter (Cobra II auto-gamma counter, Packard, USA). Experiment was performed in 2 animals. Kinetic of distribution was performed on Position emission tomography (PET) imaging, by reconstructing images from 30 minutes scans. Tumor bearing Nu/Nu Mice were imaged on a 7.5 field view LabPET8 (Gamma Medica Inc.) scan after peptide (3.7–7.4 MBq 100–200 μCi; 100 μL) administration via the caudal vein under isoflurane anaesthesia. Images were reconstructed as previously described by a three-dimensional MLEM algorithm [46] and organ-specific activity was derived and reported to injected dose per tissue volume for percentage calculation.

### *In-vivo* assays

For tumor implantation, trypsin-harvested LNCaP cell suspension was mixed with equal volume of matrigel (BD Biosciences, Bedford, MA) on ice and subcutaneously injected (200 μL) at 4 different sites (left/right hip and left/right shoulder) on Nu/Nu male mice (Charles River Laboratories, LaSalle, Canada) at density of 2 million cells/site. Tumors were periodically measured and the tumor volumes were calculated from the equation: Length•width^2^•π/6. When tumors were fully formed and palpable, mice were divided into treatment groups. For intratumoral administration of the compound, 20 μL of peptides (2.5 μg/μL) or vehicle (5% Dimethyl sulfoxide in sterile saline) was administered directly into the tumor at interval of 48 hours. For intra-venous administration experiments, 100 μL of peptide solution (2 mg/kg) or vehicle (saline) was administered through tail vein at interval of 24 hours. The total tumor volume per animal was normalized using the following formula: (total tumor volume/total tumor volume at day 27 × 100). Blood sampling (50–75 μL) from saphenous vein was performed weekly and plasma was stored at −80°C for Prostate-specific antigen (PSA) serum levels determination using a PSA EIA assay (ClinPro International, USA). Mice were housed under pathogen-free conditions and manipulations were performed in a biosafety cabinet. All experimental procedures were in accordance with the Canadian Council on Animal Care.

### Immunohistochemistry analysis

Upon sacrifice, tumors were collected and fixed in formalin and paraffin embedded. Immunohistochemistry staining was performed on 5 μM sections using standard streptavidin-biotin-peroxidase procedure with a Ventana NexEs autostainer and the solvent-resistant DAB Map Detection Kit. Antibodies (p27KIP, 1:100; Ki-67, ready to use) were purchased from Dako Canada or (PARP Asp214, 1:200) from Cell Signaling. Proliferation index was determined by the nucleus count (Ki-67^+^ cells/total cells) in sampled fields, then normalized relative to control tumor proliferation index. An average of 3–4 fields per tumor were sampled (*n* = 12 control tumors and 14 treated tumors). Immunostaining quantitation of P27^KIP^ was performed using a SuperCoolScan9000 (Nikon, USA) and Yellow channel was extracted from a CMYK color model using Fiji Software (Open Source) [47]. To avoid any off-tumor quantitation, tumors were counterstained with standard hematoxylin and eosin procedures. Apoptosis index was determined using the same method as Ki-67 counts. Pictures with 100 × and 400 × magnifications were acquired using an Axioskop2 phase-contrast microscope (Carl Zeiss, USA) and processed using ImagePro software (Media Cybernetics, USA).

## SUPPLEMENTARY METHODS, FIGURE AND TABLES



## Abbreviations

Ac: Acetyl
Amba: amidinobenzylamide
Ki: Inhibition constant
ML: Multi-Leucine peptide Ac-LLLLRVKR-NH_2_
NOTA: 1,4,7-triazacyclononane-triacetic acid
PC: Proprotein Convertase
PET: Position emission tomography
PK: Pharmacokinetic
PSA: Prostate-specific antigen
T_½_: Half-life
