# Supplementary material for: PACE4 inhibitors and their peptidomimetic analogs block prostate cancer tumor progression through quiescence induction, increased apoptosis and impaired neovascularisation
Source: Oncotarget. 2015 Feb 19;6(6):3680–93. doi: 10.18632/oncotarget.2918 (PMC4414146; doi:10.18632/oncotarget.2918)
Supplement: Supplementary file 1 [file oncotarget-06-3680-s001.pdf]

SUPPLEMENTARY METHODS

Peptide synthesis

Reagents

All amino acid derivatives and coupling reagents were purchased from ChemPep Inc. (Wellington, FL, USA) and ChemImpex International (Wood Dale, IL, USA). TentaGel S RAM resin and 2-chlorotrityl chloride resin were purchased from Rapp Polymere (Tübingen, Germany) and ChemPep Inc. (Wellington, FL, USA), respectively. 1.4.7 triazacyclononane was obtained from TCI America (Portland, OR, USA). Amba · 2HCl was synthesized as previously described [1]. Other chemicals were obtained from commercial suppliers and were used without further purification. Analytical HPLC was performed on an Agilent Technologies 1100 system equipped with a diode array detector (λ = 210, 230, and 254 nm) using an Agilent Eclipse XDB C18 column (5 µm, 4.6 × 250 mm). Semipreparative HPLC was carried out on an Agilent Technologies 1100 system using a Zorbax Eclipse XDB-C18 column (9.4 × 250 mm, 80 Å). The following solvent systems were used: [A] 0.1% aqueous trifluoroacetic acid (TFA) and [B] acetonitrile: 0.1% aqueous TFA. All peptides were obtained as TFA salts after lyophilization. Mass spectra were acquired in positive ion mode using a TripleTOF 5600.

General procedure for the peptide synthesis

The inhibitors containing the Arg residue in the P1 position were obtained manually by SPPS on a polystyrene resin – TentaGel S RAM (Rapp Polymere, 1g, capacity 0.23 mmol/g). The following amino acid derivatives were used: Fmoc-Arg(Pbf)-OH, Fmoc-Lys(Boc)-OH, Fmoc-Val-OH, Fmoc-Leu-OH, Fmoc-DLeu-OH. Amino acid coupling was performed using 3 equivalents (equivalents relative to initial loading of the resin) of Fmoc-protected amino acids, 3 equivalents of 2-(1H-7-azabenzotriazol-1-yl)-1,1,3,3-tetramethyluronium hexafluorophosphate (HATU), 3 equivalents of 1-Hydroxy-6-chloro-benzotriazole (6-Cl-HOBt) and 9 equivalents of N,N-diisopropylethylamine (DIPEA) in mixture of dimethylformamide (DMF)/ dichloromethane (DCM) (1:1 v/v). The Fmoc groups were removed by treatment with 20% piperidine in DMF. The completeness of the reaction was monitored by the Kaiser test. [2] After the final Fmoc deprotection, the N-terminal acetylation was carried out in DCM with acetic anhydride (15%) and DIPEA (15%). After completing the syntheses, the peptides were cleaved from the resin using a cocktail of trifluoroacetic acid (TFA)/triisopropylsilane(TIS)/water (95:2.5:2.5 v/v/v). Inhibitors modified with the Amba were obtained manually by a combination of SPPS and solution synthesis, as described in detail in our previous work [1].

All peptides were purified by RP-HPLC on an Agilent Technologies 1100 system using a semi-preparative Zorbax Eclipse XDB-C18 column with a linear gradient running from 25 to 50% [B] for 60 min. The pure product fractions were combined and lyophilized. Compound identification and purity was assessed by analytical HPLC (Agilent Technologies 1100 system, Waldbronn, Germany) with an Agilent Elipse XDB C18 column. High-resolution mass spectrometry (TripleTOF 5600, ABSciex, Foster City, CA) was used to confirm the identity of the pure products. According to HPLC and MS analysis, the purity of the peptides exceeded 98%. Their physicochemical properties are presented in Supporting Information Tables S1.

The NOTA-peptide conjugate was obtained on the basis of the strategy developed by Guérin et al [3] using a combination of the solid phase approach and solution synthesis, as described in Scheme S1. The P8-P2 peptide segment was synthesized manually *via* SPPS on a 2-chlorotrityl-chloride resin using the Fmoc strategy. The first amino acid derivative was assembled on the resin as follows: Fmoc-Lys(Boc)-OH (1.2 equiv) was dissolved in dry DCM (15 mL) and DIPEA (4 equiv) and was immediately added to the resin (1 g, 1 equiv, resin loading: 0.8 mmol/g). After 120 min of shaking at room temperature (RT), the resin was treated with DCM/methanol(MeOH)/DIPEA (17:2:1 v/v/v), washed several times with DCM, DMF, and DCM, and dried in *vacuo*. The loading of the resin was ~0.4 mmol/g. The synthesis of the NOTA-peptide conjugate was performed on a scale of 200 µmol. The remaining amino acids were incorporated according to standard coupling procedures using 3 equivalents of Fmoc amino acids, HATU with 6-Cl-HOBt as a coupling agent and DIPEA (9 equiv) in anhydrous DMF. The Fmoc groups were removed by treatment with 20% piperidine in DMF. After the final Fmoc deprotection, the bromoacetic acid activated as the symmetric anhydride was coupled to the peptide. To the solution of the bromoacetic acid (5 equiv) in anhydrous DCM at 0°C N,N'-diisopropylcarbodiimide (DIC, 5 equiv) was added. The resulting mixture was stirred at 0°C for 15 minutes, and then was diluted with anhydrous DMF (DCM/DMF, 1/1, v/v) and added to the resin pre-swelled with DCM. After 60 min of coupling, the resin was washed several times with DMF, MeOH, DMF, MeOH and DCM. Next, the bromoacetylated peptidyl-resin was treated with a solution of 1.4.7 triazacyclononane (5 equiv) in anhydrous DCM for 3 hours in RT. After the coupling, the resin was washed several times with dimethyl sulfoxide (DMSO), DMF, MeOH and DCM. The cyclen peptidyl resin was then alkylated with *tert*-butyl 2- bromoacetate (3 equiv) in the presence of DIPEA (3 equiv) in anhydrous DMF. The protected peptide was cleaved from resin by treatment with HFIP/DCM (1:4 v/v) for 2 hours at

RT. The solvent was removed in *vacuo*, and the peptide was lyophilized from 50% t-butanol in water. Protected peptide (1 equiv) and Amba · 2HCl (2 equiv), COMU (2 equiv), and N-methylmorpholine (NMM, 4 equiv) were dissolved in DMF and stirred for 4 hours at RT. The solvent was removed in *vacuo* to give a brownish oil, which was then dissolved in TFA/TIS/H_2O (95:2.5:2.5 v/v/v), stirred for 3 hours at RT, precipitated by cold ether, washed two times with ether, and lyophilized. The crude peptide was purified by RP-HPLC.

Mass spectrometry analyses for pharmacokinetic assays

After acetonitrile precipitation and filtration of collected plasma, sample were analysed at PhenoSwitch Bioscience (Sherbrooke, Canada) by LC-MS/MS on a TripleTOF 5600 mass spectrometer (ABSciex; Foster City, CA) equipped with DuoSpray source. Sample where introduce to the ESI source in a 25um ESI probe (Eksigent) using a microLC200 system (Eksigent) equipped with a 100 mm × 50 mm HALO C18 2.7μm column (Eksigent). Samples were injected by overfilling a 5 uL injection loop and compounds where separated on gradient of water containing 0.2% formic acid and 3% DMSO (A) and Acetonitrile containing 0.2% formic acid and 3% DMSO (B). Column temperature was set at 50°C. The 6 minutes gradient at 20μL/min was the following: hold at 10% B for 0.6 min, gradient from 10% B to 60% from 0.6 min to 3.8 min, hold at 90% B from 3.8 to 4.9 min and equilibration from 5 to 6 min at 10% B. Source parameters where the following: curtain gas was set at 27, gas 1 was set at 15, gas 2 was set at 20, ion source voltage was set at 5500, ESI probe temperature was set at 225, declustering potential was set at 100v. Compounds were monitored using optimized collision energy parameter in product ion mode (Supplementary Table S2) with a mass range from 100 to 1200 m/z. A standard curve of peptide Ac-[DLeu]LLLRVK-Amba with concentration ranging between 25 to 600 ng/mL was prepared in commercial plasma (Novi, MI, USA). Analysis was performed using MultiQuant software V2.0 using signal finder algorithm (ABSciex, Foster City, CA) by selecting the appropriate product ion transition (Supplementary Table S3) with a selection window of 0.05Da and pharmacokinetic parameters were calculated using PKSolver [4]. Pharmacokinetic profile was calculated for every animal and data in the table are mean and standard deviation of calculated parameters.

Abbreviations

6-Cl-HOBt, 1-hydroxy-6-chloro-benzotriazole, Ac – acetyl, Amba – 4-amidinobenzylamide, COMU – 1-Cyano-2-ethoxy-2-oxoethylidenaminooxy)dimethylamino-morpholino-carbeniumhexafluorophosphate, DCM – dichloromethane, DIC – N,N'-diisopropylcarbodiimide, DIPEA – N,N-diisopropylethylamine, DMF – dimethylformamide, DMSO – Dimethyl sulfoxide, HATU – 2-(1H-7-azabenzotriazol-1-yl)-1,1,3,3-tetramethyluronium hexafluorophosphate, MeOH – methanol, NMM – N-methylmorpholine, RT, room temperature, SPPS, solid-phase peptide synthesis, TFA – trifluororacetic acid; TIS; triisopropylsilane.

REFERENCES FOR SUPPORTING INFORMATION

1. Kwiatkowska A, Couture F, Levesque C, Ly K, Des jardins R, Beauchemin S, Prahl A, Lammek B, Neug ebauer W, Dory YL, Day R. Design, Synthesis, and Structure-Activity Relationship Studies of a Potent PACE4 Inhibitor. Journal of medicinal chemistry. 2014; 57:98–109.
2. Kaiser E, Colescott RL, Bossinger CD, Cook PI. Color test for detection of free terminal amino groups in the solid-phase synthesis of peptides. Anal Biochem. 1970; 34:595–598.
3. Guerin B, Ait-Mohand S, Tremblay MC, Dumulon-Perreault V, Fournier P, Benard F. Total solid-phase synthesis of NOTA-functionalized peptides for PET imaging. Organic letters. 2010; 12:280–283.
4. Zhang Y, Huo M, Zhou J, Xie S. PKSolver: An add-in program for pharmacokinetic and pharmacodynamic data analysis in Microsoft Excel. Computer methods and programs in biomedicine. 2010; 99:306–314.

SUPPLEMENTARY FIGURE AND TABLES

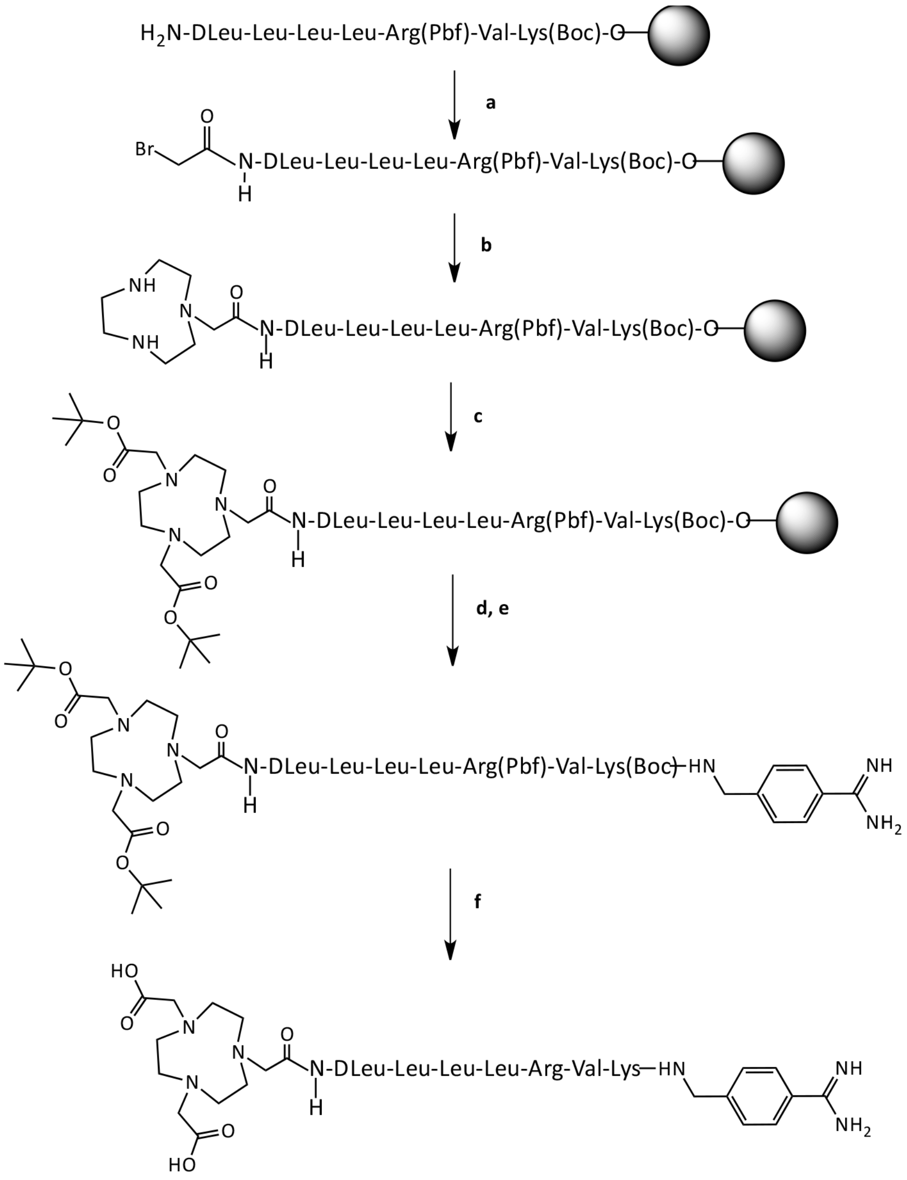

Supplementary Figure S1: Synthesis of the NOTA-[DLeu]LLLRVK-Amba. The P8-P2 segment was prepared on a 2-chlorotrityl chloride resin. **(A)** 5 equiv of bromoacetic acid, 5 equiv of DIC, anhydrous DCM/DMF, (1/1, v/v), 0°C, 60 min; **(B)** 5 equiv of 1.4.7 triazacyclononane, anhydrous DCM, 3h; **(C)** 2 equiv of *tert*-butyl 2- bromoacetate, 3 equiv of DIPEA, anhydrous DMF, 2h; **(D)** hexafluoroisopropanol /DCM (1/4, v/v), 1.5 h; **(E)** 2 equiv of Amba • 2 HCl, 2 equiv of COMU and 4 equiv of NMM, DMF, 4 h; **(F)** TFA/TIS/H_2O (95/2.5/2.5, v/v/v), 2 h.

Supplementary Table S1: Physicochemical properties of the peptides used in this study

Peptide	HPLC[a] (T^R)	MS calculated	*(M+2H)*$^{2+}$	*(M+3H)*$^{3+}$
Ac-LLLLRVKR-NH_2	21.087	1051.37	526.37	351.25
Ac-[DLeu]LLLRVKR-NH_2	21.450	1051.37	526.37	351.25
Ac-LLLLRVK-Amba	23.848	1027.35	514.36	343.24
Ac-[DLeu]LLLRVK-Amba	22.321	1027.35	514.36	343.24
NOTA-[DLeu]LLLRVK-Amba	19.589	1270.61	635.59	423.60

[a] linear gradient from 10 to 70% of [B] in [A] for 50 min, AGILENT Elipse XDB C_{18} column; [A] 0.1% aqueous trifluoroacetic acid (TFA), [B] acetonitrile: 0.1% aqueous TFA.

Supplementary Table S2: Instrument parameters used for pharmacokinetic studies using mass spectrometry

Peptide	Product ion mass	Cycle time (ms)	Declustering potential	Colision energy	Ion release delay	Ion release width
Ac-LLLLRVKR-NH_2	351.30	325	100	18	73	28
Ac-[DLeu] LLLRVK-Amba	343.25	325	100	20	72	27

Supplementary Table S3: Quantification parameters used for pharmacokinetic studies using mass spectrometry

Peptide	Extracted mass (m/z)	Expected RT	Units
Ac-LLLLRVKR-NH_2	654.45<br>448.82	2.31	ng/mL
Ac-[DLeu]LLLRVK-Amba	630.42<br>436.80	1.93	ng/mL
